# Plant-parasitic nematodes: towards understanding molecular players in stress responses

**DOI:** 10.1093/aob/mcw260

**Published:** 2017-01-12

**Authors:** François-Xavier Gillet, Caroline Bournaud, Jose Dijair Antonino de Souza Júnior, Maria Fatima Grossi-de-Sa

**Affiliations:** 1 Embrapa Genetic Resources and Biotechnology, PqEB Final Av. W/5 Norte, CEP 70·770-900, Brasília, DF, Brazil; 2 Catholic University of Brasilia, Brasília-DF, Brazil

**Keywords:** MAMP- and PAMP-triggered immunity, oxidative burst, reactive species, phytoalexins, plant parasitic nematodes, DAF-16/FoxO, SKN-1/Nrf2, cytoprotective mechanisms, insulin/IGF-1, DAF pathway, dauer

## Abstract

**Background** Plant–parasitic nematode interactions occur within a vast molecular plant immunity network. Following initial contact with the host plant roots, plant-parasitic nematodes (PPNs) activate basal immune responses. Defence priming involves the release in the apoplast of toxic molecules derived from reactive species or secondary metabolism. In turn, PPNs must overcome the poisonous and stressful environment at the plant–nematode interface. The ability of PPNs to escape this first line of plant immunity is crucial and will determine its virulence.

**Scope** Nematodes trigger crucial regulatory cytoprotective mechanisms, including antioxidant and detoxification pathways. Knowledge of the upstream regulatory components that contribute to both of these pathways in PPNs remains elusive. In this review, we discuss how PPNs probably orchestrate cytoprotection to resist plant immune responses, postulating that it may be derived from ancient molecular mechanisms. The review focuses on two transcription factors, *DAF-16* and *SKN-1*, which are conserved in the animal kingdom and are central regulators of cell homeostasis and immune function. Both regulate the unfolding protein response and the antioxidant and detoxification pathways. DAF-16 and SKN-1 target a broad spectrum of *Caenorhabditis elegans* genes coding for numerous protein families present in the secretome of PPNs. Moreover, some regulatory elements of DAF-16 and SKN-1 from *C. elegans* have already been identified as important genes for PPN infection.

**Conclusion** *DAF-16* and *SKN-1* genes may play a pivotal role in PPNs during parasitism. In the context of their hub status and mode of regulation, we suggest alternative strategies for control of PPNs through RNAi approaches.

## INTRODUCTION

Plants have evolved immune defence mechanisms against pathogens that employ two different detection systems. As summarized in the classic zig-zag model, defence is based on (1) perception of conserved microbial-associated molecular patterns (MAMPs) or pathogen-associated molecular patterns (PAMPs) by cell surface-localized pattern recognition receptors (PRRs) that initiates basal immunity known as MTI/PTI (MAMP- or PAMP-triggered immunity) and (2) recognition of pathogenic effectors by intracellular nucleotide-binding domain leucine-rich repeat proteins (NB-LRRs) leading to effector-triggered immunity (ETI) (Jones and Dangl, 2006; Dodds and Rathjen, 2010). Although our knowledge of immune signalling components is currently poor with regard to plant/nematode interactions, molecular mechanisms of plant defence imply a certain degree of conservation among a broad range of studied pathosystems (Goverse and Smant, 2014; Zipfel, 2014; Holbein *et al.*, 2016). During the early stages of infection, plant recognition of plant-parasitic nematodes (PPNs) leads rapidly to the production of reactive species [reactive oxygen (ROS) and reactive nitrogen (RNS)] and toxic molecules derived from secondary metabolism (Melillo *et al.*, 2011). This toxic environment induced by the host plant results in oxidative stress in the animal. Through comparison with the *Caenorhabditis elegans* model system, we postulate that PPNs orchestrate an adapted response against the stressful conditions imposed by plant immunity.

In free-living nematodes, the dauer stage refers to an arrested developmental variant that circumvents harsh environmental conditions (Hu, 2007; Perry *et al.*, 2009; Crook, 2014). Interestingly, dauer larvae share similarities with infective juvenile formation or pre-parasitic stage parasitic nematodes (Hu, 2007; Dieterich and Sommer, 2009; Davies and Curtis, 2011; Crook, 2014; Sommer and Mayer, 2015). For instance, pre-parasitic plant nematodes show certain identical morphological and metabolic features, such as a strong cuticle and fat storage (Cassada and Russell, 1975; Robinson *et al.*, 1987; Davies and Curtis, 2011). Another relevant similarity is the high resistance of PPNs to oxidative stress (Larsen, 1993; Dubreuil *et al.*, 2011; Vicente *et al.*, 2015). With regard to the evolutionary history of nematodes, Blaxter and Koutsovoulos (2014) proposed that the transition from a free-living habit to parasitism defined three origins of plant parasitism in the phylum Nematoda. Interestingly, Sommer and Mayer (2015) have postulated that dauer formation might be a pre-adaptation that drove nematodes to phoresy, necromeny and then parasitism (Dieterich and Sommer, 2009; Crook, 2014). Morphological and physiological characteristics of dauer larvae have been seen as evolutionary catalysts that give rise to the predisposition of nematodes to withstand harsh conditions. Such an environment is met by the parasitic nematode in direct contact with the arsenal of toxic molecules produced by the host immune system. Comparative genomics using C. *elegans* and PPNs have enabled the identification of *C. elegans DAF* (dauer abnormal formation) orthologues in different clades of PPNs (McCarter *et al.*, 2003; Abad *et al.*, 2008; Opperman *et al.*, 2008; Ogawa *et al.*, 2009; Dieterich and Sommer, 2009; Crook, 2014; Cotton *et al.*, 2014; Burke *et al.*, 2015). Moreover, the characterization of some essential *DAF* genes has been published for other free-living, necromenic and animal-parasitic nematodes (APNs), supporting the central role of these genes in any nematode lifestyle (Birnby *et al.*, 2000; Ogawa *et al.*, 2009; Bento *et al.*, 2010; Sommer and Mayer, 2015; Albarqi *et al.*, 2016).

In this review, we provide an overview of the molecular interplay between plants and nematodes, with particular attention given to the early stages of infection. In the first section, we describe one component of plant immunity that allows the production of reactive species and toxic metabolites in response to nematode intrusion. In the second section, we report on whether certain genes play pivotal and conserved roles in PPN stress responses during plant parasitism. Finally, the review describes the potential function of *C. elegans* DAF-16 and SKN-1 transcription factor genes involved in adaptative responses to environmental stresses through three essential pathways, namely the antioxidant pathway, the detoxification pathway and the unfolding protein response (UPR). Conserved regulatory components of DAF-16 and SKN-1 in PPNs and modes of action are discussed.

## SECTION I: PLANT BASAL DEFENCE, THE APOPLAST AND THE REDOX BALANCE

### Early perception of nematode intrusion

PPNs have evolved sophisticated strategies to overcome plant innate immunity, to mitigate host cell damage, and to promote feeding site development and reproduction (Gheysen and Mitchum, 2011). The recent review by Goverse and Smant (2014) highlighted that PPNs must address critical developmental transitions in plant parasitism, including (1) host synchronization, (2) host attraction, (3) host invasion, (4) migration inside the host, (5) initiation of a permanent feeding structure, (6) expansion of a permanent feeding structure and (7) maintenance of a transfer cell-like function. These authors note that several sequential ‘go/no-go checkpoints’ during the plant–nematode interaction underpin a complex and dynamic interplay. Interestingly, fine-tuned and coordinated response strategies occur throughout plant–pathogen interactions, and the interactions are constantly evolving within specific spatio-temporal and environmental phenomena (Thomma *et al.*, 2011; Pritchard and Birch, 2014; Andolfo and Ercolano, 2015). To date, the later events of PPN invasion in host root tissues have been extensively studied, i.e. when the nematode becomes sedentary, and plant defence is widely suppressed (reviewed by Goverse and Smant, 2014). Nevertheless, it would be noteworthy to provide investigations of oxidative stress responses from the very moment that PPNs invade the host, to elucidate the molecular interaction between the nematode and host plant at early parasitism. More recently, Manosalva *et al.* (2015) showed that PPNs secrete conserved molecules, so-called ascarosides, eliciting MAMP responses at nanomolar levels in various plants. The ascarosides represent a class of pheromones exclusively identified in the phylum Nematoda (Choe *et al.*, 2012*a*). Similar to other known MAMPs such as flagellin (flg22) and chitin, ascaroside perception triggers an enhanced microbial-associated molecular-patterns-triggered immunity (MTI) response against a broad spectrum of pathogens (Manosalva *et al.*, 2015). Even if ascarosides are proposed to be MAMPs from PPNs, their cognate PRRs have not yet been identified. It was shown that some ascarosides are continuously secreted by nematodes (Noguez *et al.*, 2012; Schroeder, 2015), suggesting that ascarosides might be diffused in the rhizosphere and can trigger plant defence responses before physical contact. In addition, PPN entry into root tissues is facilitated by mechanical force or the release of enzymes that alter cell-wall integrity (by cell-wall-degrading enzymes (CWDEs) (Bellincampi *et al.*, 2014; Bohlmann and Sobczak, 2014). Comparing genomic and transcriptomic approaches across distant PPN species, Rai *et al.* (2015) provided a comprehensive view of the CWDEs produced by nematodes from initial to late infectious stages. Nematode intrusion into a host cell may also activate the production of damage-associated molecular patterns (DAMPs) (Haegeman *et al.*, 2011; Mitchum *et al.*, 2013) that induce several downstream signalling events in plant immunity (Boller and Felix, 2009; Heil and Land, 2014). However, DAMPs have not yet been identified in plant–nematode interactions. Although the activation of basal defence responses remains underexplored, numerous reviews have shed light on their relevance to identify key molecular players that are involved at early stages of infection (Feng and Shan, 2014; Holbein *et al.*, 2016).

### The toxic cocktail of plant immunity

Plant defence responses trigger secondary metabolism towards the synthesis of toxic molecules, i.e. phytoalexins that belong to a class of low-molecular-mass secondary metabolites (Chitwood, 2002; Ahuja *et al.*, 2012). The secretion of phytoalexins is also correlated with the formation of root border cells when the root tips are exposed to a plant pathogen (Cannesan *et al.*, 2011; Baetz and Martinoia, 2014). Investigation of root infection in banana (*Musa* spp.) by the burrowing nematode *Radopholus similis*, for example, revealed local induction and accumulation of phenalenone-type phytoalexins, which are derived from the phenylpropanoid pathway (Hölscher *et al.*, 2014). Similarly, production and exudation of phytoalexins in soybean root tissues infected with the cyst nematode (CN) *Heterodera glycines* are also restricted to resistant cultivars (Huang and Barker, 1991). Overexpression of the Arabidopsis phytoalexin-deficient 4 gene (*AtPAD4*), a lipase-like protein involved in plant defence, promoted by salicylic acid (SA) and phytoalexins, enhances resistance in soybean roots in response to PPN species *Meloidogyne incognita* (root-knot nematode, RKN) and *Heterodera glycines* (CN) (Youssef *et al.*, 2013). An increased level of phytoalexins thus helps to induce the plant defence machinery following PPN attack. Increased understanding of their toxic activity and species-specific responses will benefit the development of disease control strategies.

Although plants produce a combination of toxic molecules that comprise a large variety of secondary metabolites, they also release reactive species. During nematode invasion, the plant generates an unfavourable oxidative environment to the parasite that plays a pivotal role in the MTI and ETI defence signalling pathways, triggering ROS accumulation through host immunity (Torres *et al.*, 2006). The host plant senses and finely adapts its cellular redox status by activating the antioxidant pathway, which also occurs in an NPR1 (non-expressor of pathogenesis-related 1)-dependent manner (Després *et al.*, 2003; Mou *et al.*, 2003). Regulation of the antioxidant pathway enables the expression of genes encoding ROS-scavenging enzymes (Apel and Hirt, 2004; Foyer and Noctor, 2005), preventing intracellular oxidative damage to the host while inducing oxidative stress in the pathogen inside the host apoplast (Delaunois *et al.*, 2014). Oxidative stress is also sensed and orchestrated by NPR1, which acts as a key regulator in plant defence responses, including the SA pathway (Pieterse and Van Loon, 2004; Siddique *et al.*, 2014). NPR1 overexpression increases MTI and ETI responses, and is associated with a decrease in the number of galls and egg masses in response to *M. incognita* infection (Priya *et al.*, 2011).

During the early stage of infection, the perception of PPNs triggers an oxidative burst in root tissues. At the plant cell surface, two major oxidant enzyme families govern the oxidative burst responses, corresponding to the plasma membrane-localized nicotinamide adenine dinucleotide phosphate (NADPH) oxidases (NOXs, which are also called respiratory burst oxidases, or Rbohs) and/or the cell-wall-localized (or apoplastic) peroxidases (Lambeth, 2004; Mittler *et al.*, 2011; Siddique *et al.*, 2014). In addition, other oxidative enzymes are also implicated in the production of reactive species, such as xanthine oxidases, oxalate oxidases and amine oxidases (Wojtaszek, 1997; Hewezi *et al.*, 2010; Moschou and Roubelakis-Angelakis, 2014; Iberkleid *et al.*, 2015). Together, these enzymes contribute to the generation of reactive species, with distinct roles in MTI- and ETI-signalling responses (Daudi *et al.*, 2012; Considine *et al.*, 2015). Moreover, both the spatio-temporal aspect and the intensity of ROS released are relevant in the immune response, with the plant immune system generally governed by biphasic ROS production*.* At a first level, ROS are produced at low concentration in plant cells, which can then be followed by a second level of synthesis with much higher concentrations. The latter level also leads to the local self-sacrifice of a few plant cells, known as the hypersensitive response (HR) or cell death (Levine *et al.*, 1994; Foyer and Noctor, 2005). The HR is particularly toxic for microbial invaders, in incompatible interactions, as H_2_O_2_ reaches an estimated concentration of 5–10 mm (Levine *et al.*, 1994; Desikan *et al.*, 1998).

Furthermore, the intensity and spatio-temporal nature of ROS release are also strongly dependent on the host genotype, nematode pathotype (virulent or avirulent), and environmental conditions which can determine the compatibility of the interaction (Bhattacharjee, 2012; Goverse and Smant, 2014; Feng and Shan, 2014). For example, strong oxidative bursts have been identified in early responses of wheat cultivars in incompatible interactions with *Heterodera avenae*, which are correlated with the up-regulation of several apoplastic peroxidases (Simonetti *et al.*, 2009; Kong *et al.*, 2015). The penetration of tomato root tissues by virulent or avirulent RKN populations generates the production of reactive species in a local and rapid manner. Indeed, the capacity of the plant to induce the HR can also be pathotype-dependent. For example, whilst the HR has been observed in *Mi*-resistant plants challenged with avirulent PPN (incompatible interaction), virulent pathotypes can overcome MTI, without triggering the HR, and develop feeding sites (compatible interaction) (Waetzig *et al.*, 1999; Melillo *et al.*, 2006, 2011). Interestingly, Siddique *et al.* (2014) showed a positive role for H_2_O_2_ in Arabidopsis susceptibility to cyst nematodes, indicating that nematodes can also induce ROS to promote infection. Correlations between nematode virulence and resistance to oxidative stress have been observed in the pinewood nematode, *Bursaphelenchus xylophilus* (Vicente *et al.*, 2015). Together, these findings support the idea that the establishment of a cytoprotective mechanism against environmental oxidative stresses is a crucial prerequisite for successful PPN infection at both early and later time points after contact with the plant cell.

In general, reactive species primarily trigger two biological effects. First, they may create a cytotoxic environment for PPNs. For example, the superoxide anion ($O_{2}^{-}$) is highly damaging and diffuses over a short distance, whereas hydrogen peroxide (H_2_O_2_) is less reactive but can cross cell membranes and diffuses into the cell (Winterbourn, 2008). Secondly, ROS, such as hydrogen peroxide, and RNS, such as nitric oxide (NO), also act as signalling molecules at local and systemic levels, thereby influencing many cellular processes (Mittler *et al.*, 2011; Suzuki *et al.*, 2011; Considine *et al.*, 2015; Sandalio and Romero-Puertas, 2015). Interestingly, RKN infection is not sufficient to trigger ROS in *Rk*-resistant cowpea plants and leads to a delayed defence response without an obvious HR (Das *et al.*, 2008). These authors speculate that PPNs may alleviate or neutralize ROS release from the host plant to avoid localized cell death, through the manipulation of plant ROS-scavenging enzymes (Das *et al.*, 2010). In fact, it was shown that *Heterodera schachtii* infection activates plant NOXs in plants to stimulate a local ROS production; subsequently, the release triggers antioxidant pathways (Siddique *et al.*, 2014). Another study reported the discovery of a secretory effector (10A06) from *Heterodera glycines* to support this hypothesis (Hewezi *et al.*, 2010). The *Heterodera schachtii* 10A06-homologue effector also interacts with Arabidopsis spermidine synthase 2 (SPDS2), which increases the activity of polyamine oxidase (PAO) and consequently enhances the antioxidant pathway in host plants (Hewezi *et al.*, 2010). In contrast, PAO overexpression in *Nicotiana tabacum* plants leads to a greater H_2_O_2_ accumulation that is associated with disease tolerance to bacteria and oomycetes (Moschou *et al.*, 2009; Moschou and Roubelakis-Angelakis, 2014). It seems that PAO enzymatic activity plays a pivotal role in cellular redox homeostasis in response to pathogen infection. Following these findings, it was proposed that PPNs might modulate the production of reactive species, not as toxic compounds, but as signalling molecules to activate antioxidant pathways (Goverse and Smant, 2014; Holbein *et al.*, 2016).

The role of reactive species as signalling molecules is also crucial in the animal cell, particularly in those in which an oxidative stress response is implicated (Lambeth, 2004; Nathan and Cunningham-Bussel, 2013). Given the proximity of the PPN epidermis to the cell wall, H_2_O_2_ can diffuse across the nematode cell membrane and thus influences the redox cellular homeostasis of the animal. By extension, the plant cellular redox homeostasis can influence the PPN redox homeostasis and PPN behaviour during parasitism. The plant redox homeostasis perception by PPNs could be considered as a parameter integrated to the ‘go/no-go checkpoints’ model (Goverse and Smant, 2014). In the second section, we will discuss the potential signalling pathway involved in ROS perception by PPNs.

Extensive evidence has shown that ectoparasitic and endoparasitic PPNs employ sophisticated adaptation mechanisms related to their mode of parasitism (Evans and Perry, 2009; Dieterich and Sommer, 2009; Crook, 2014). We previously stated that PPNs are confronted with a hostile environment from the first contact with the host cell wall. Indeed, the oxidative burst in plant occurs at early stages of PPN infection. Most proteome and transcriptome analyses have reported that PPNs evade reactive species in the apoplast through the release of ROS scavengers, glutathione peroxidase (GPx), peroxidase (PER), peroxiredoxin (PRXs) and catalases (CTLs) (Bellafiore *et al.*, 2008; Dubreuil *et al.*, 2011; Shinya *et al.*, 2013). We will provide an overview on this point in the second section. PPNs also secrete effectors to enable the control of cellular signalling pathways and thus to stabilize long-term relations with their host plants (Bellafiore and Briggs, 2010; Kyndt *et al.*, 2013; Mantelin *et al.*, 2015). In addition to Hs10A06 protein, two other nematode effectors have been shown to fine-tune oxidative stress responses in the host plant. The first example implicates nematode effectors Hs4F01 and Hg4F01 identified from two CNs, *Heterodera schachtii* and *Heterodera glycines.* These 4F01 effectors are secreted proteins similar to annexins in plants (Gao *et al.*, 2003; Patel *et al.*, 2010). Clark *et al.* (2010) have proposed that Arabidopsis annexins participate in the oxidative stress response. Hs4F01 overexpression in Arabidopsis plants is beneficial to PPN parasitism, unlike the RNAi-mediated knockdown effect. Consequently, the interaction of 4F01 with an Arabidopsis oxidoreductase suggests that this effector may be implicated in the regulation of oxidative stress responses in the host plant. The second example concerns the transthyretin-like (TTL) protein that is secreted by *Meloidogyne javanica*, which is likely to occur during early stages of infection*.* The Mj-TTL5 effector interacts with a ferredoxin: thioredoxin reductase catalytic subunit (AtFTRc) from Arabidopsis. This interaction is correlated with ROS scavenging and plant susceptibility against PPNs. Because ROS, particularly H_2_O_2_, are signalling molecules in plant immunity, their suppression is associated with the attenuation of host resistance to nematode infection (Lin *et al.*, 2016). Notably, both PPN effectors (annexin-like and transthyretin-like effectors) are derived from ancient protein families that are conserved in the plant and animal kingdoms, with their functions often associated with oxidative stress (Richardson and Cody, 2009; Clark *et al.*, 2010; Lauritzen *et al.*, 2015).

Whilst ROS-scavenging enzymes act as cytoprotective determinants, effectors may also play a key regulatory role in plant signalling pathways. Most investigations to date on the plant–nematode interaction have focused on how PPN effectors modulate these pathways. Although rarely discussed, understanding that PPNs may also struggle against the plant oxidative stress responses appears essential. To face this stressful environment, PPNs may imply a complex orchestration of several cellular signalling pathways in different organs within these nematodes. In the following section, we raise questions about how PPNs manage the oxidative stress response and which molecular players may be determinants of this process.

## SECTION II: POTENTIAL KEY REGULATORS OF OXIDATIVE STRESS RESPONSES IN PPNs

### DAF-16 and SKN-1: from the *C. elegans* model system to PPNs

#### DAF-16 and SKN-1 in the *C. elegans* oxidative stress response.

The generation of *daf* mutants in *C. elegans* in conjunction with epistasis analysis and genetic population studies revealed various key genes in the DAF pathway (Fielenbach and Antebi, 2008). Consequently, numerous *daf* orthologue genes have been identified in the phylum Nematoda. For example, the *DAF-12* gene encodes a nuclear receptor acting at the crossroads of important pathways, such as entry/exit regulation of the dauer formation. These pathways have also been implicated in development, metabolism and behaviour in other phylogenetically distant free-living nematodes, APNs (Ogawa *et al.*, 2009; Bento *et al.*, 2010; Albarqi *et al.*, 2016) and in the PPN *B. xylophilus* (Zhao *et al.*, 2013). Other genes from the DAF pathway have been reported to be necessary for parasitism, such as *DAF-21*/*HSP90* (Birnby *et al.*, 2000; Gillan *et al.*, 2009; Lourenço-Tessutti *et al.*, 2015). This finding raises the question of whether other *DAF* genes are determinants at different steps during the parasitic nematode life cycle.

**Fig. 1.**
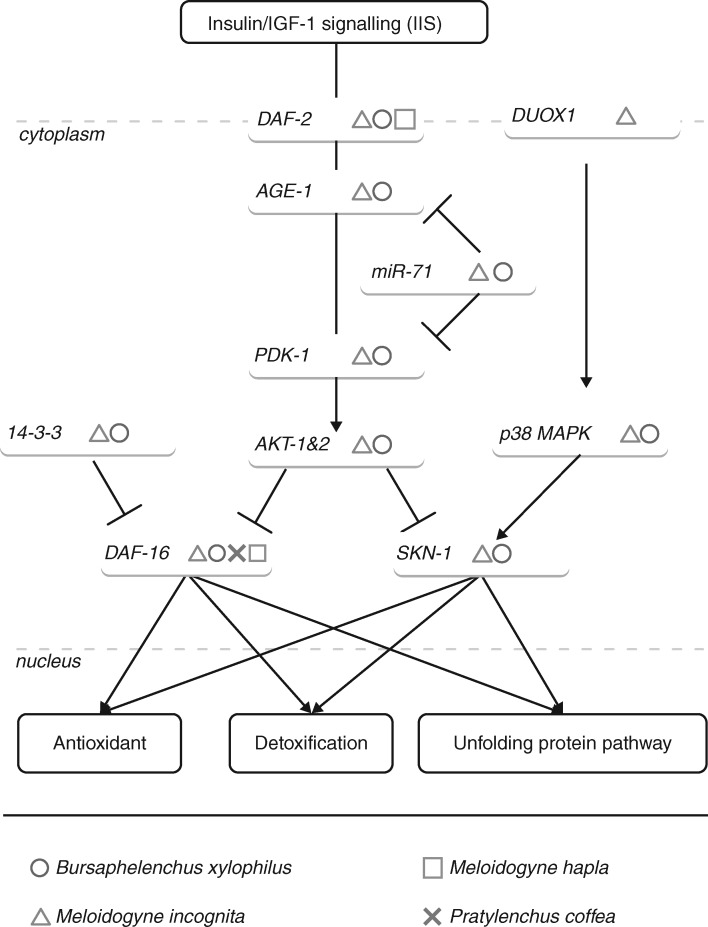
Regulatory components of DAF-16 and SKN-1 identified in plant-parasitic nematodes. DAF-2 is the insulin receptor during IIS signaling. In *C. elegans*, IIS signalling activates the phosphorylation cascade of PDK-1/AKT, leading to the sequestration of DAF-16 and SKN-1 in the cytoplasm. miR-71 expression inhibits the phosphorylation cascade, allowing the translocation of DAF-16 and SKN-1 to the nucleus. The genes identified for *B. xylophilus*, *M. incognita*, *M. hapla* or *P. coffea* are tagged with a dedicated symbol (see key). The most relevant regulatory components of *DAF-16* and *SKN-1* are highlighted here according to the identification of the orthologous genes between plant-parasitic nematodes and *C. elegans*.

Hence, an intimate connection between the *C. elegans* dauer stage and DAF pathway and oxidative stress responses has been described, supported by a remarkable level of resistance to hydrogen peroxide in the dauer stage, which is 20-fold higher than observed in the nematode adult stage (Larsen, 1993). Similarly, nematode J2s that have not yet entered the root (pre-parasitic plant nematodes) also provide pronounced resistance to oxidative stress, correlating with their degree of virulence (Dubreuil *et al.*, 2011; Vicente *et al.*, 2015; Li *et al.*, 2016). In fact, previous findings have reported the insulin/IGF-1 signalling (IIS) pathway as a mediator between development and stress responses following the model ‘*should I stay or should I go*’ (Schindler and Sherwood, 2014). Specifically, the IIS pathway modulates oxidative stress responses in nematodes at several levels and acts more generally in animals (McElwee *et al.*, 2004; Fielenbach and Antebi, 2008; Kenyon, 2010; Murphy and Hu, 2013). Activation of the dauer formation relies on the removal of the DAF pathway repression that is mediated by IIS (Matyash *et al.*, 2004) (Fig. 1). In *C. elegans*, null mutations in *daf-2*, a membrane receptor for insulin, led to a constitutive dauer formation and enhanced resistance to oxidative stress in animals (Larsen, 1993; Honda and Honda, 1999). Two key regulators of the oxidative stress response, SKN-1 and DAF-16, are negatively regulated by DAF-2 and, thus, by the IIS pathway. Remarkably, the IIS pathway is distinct from the DAF pathway. Moreover, even if both SKN-1 and DAF-16 are regulated by DAF-2 and respond to environmental stresses, only DAF-16 is implicated in dauer entry (Tullet *et al.*, 2008; Ewald *et al.*, 2015). The remaining question to be considered is whether *SKN-1* and *DAF-16* are conserved functional genes in plant nematode parasitism.

#### Search for DAF-16 and SKN-1 orthologues in PPNs.

The free radical theory initially postulated that ageing is associated with an increased intracellular accumulation of ROS (Beckman and Ames, 1998; Shore and Ruvkun, 2013). Likewise, the oxidative resistance of a *C. elegans* mutant in the IIS was associated with lifespan extension (two- to three-fold), generating a rising interest in this pathway. DAF-16 was identified as a core component in the management of oxidative stress (Murakami and Jonhson, 2001). In relation to parasitic nematodes, combined *in silico* and transcriptional analyses have shown the presence of *C. elegans DAF-16* orthologous genes in APNs from the genera *Strongyloides* (Massey *et al.*, 2003; Hu *et al.*, 2010; Hunt *et al.*, 2015) and *Ancylostoma* (Gao *et al.*, 2009, 2015). Whole genome sequence data for PPNs has also revealed that the *DAF-16* orthologue gene from *C. elegans* is extended to *Meloidogyne hapla* (Opperman *et al.*, 2008), *Pratylenchus coffea* (Burke *et al.*, 2015), *B. xylophilus* (Kikuchi *et al.*, 2011) and *M. incognita* (Abad *et al.*, 2008).

DAF-16 is a forkhead class transcription factor, homologous to the human FoxO (Forkhead box O) protein family and also conserved across animal species (Obsil and Obsilova, 2011; Nakagawa *et al.*, 2013, Webb *et al.*, 2016). *In silico* protein sequence analysis based on the winged DNA-binding domain (DBD) allowed potential orthologues to be identified in PPNs (Table 1). The high degree of conservation for its DBD may be explained by its high connectivity within several signalling pathways (Shimeld *et al.*, 2010). The decoding of SKN-1 function with regard to stress responses and ageing has also been studied (Blackwell *et al.*, 2015). Futhermore, SKN-1 is also well conserved in animals and represents a functional orthologue of the human nuclear transactivator Nfr2 (NF-E2-related factor), a major regulator of oxidative stress responses (Kensler *et al.*, 2007) and other signalling pathways, including developmental pathways (Hayes and Dinkova-Kostova, 2014). Conservation of the SKN-1 regulatory pathway was confirmed following the sequencing of the genome for the PPN *B. xylophilus* (Kikuchi *et al.*, 2011). Notably, Choe *et al.* (2012*b*) revealed that SKN-1 is generally well conserved among PPNs and APNs. We extended the search for SKN-1 in PPNs through BLAST searches by using *C. elegans* SKN-1 as the query protein sequence (Table 2). Like DAF-16, SKN-1 is conserved at its DNA-binding motif, which is composed of a CNC (Cap-n-Collar) and BR (Basic Region) (Rupert *et al.*, 1998; Choe *et al.*, 2012*b*). From the available PPN genomic resources we outlined that DAF-16 and SKN-1 are conserved upon the presence of predicted DBD in PPNs.

**Table 1:** Homology searches of DAF-16 in the phylum Nematoda for its respective core DNA-binding regions

Species	Nematode clade	Current annotation	Core DNA binding region	Website/Reference
*Caenorhabditis elegans*	V	O16850	_222_AGWK**NS**I**RH**NL**SL**HSRF_238_	Massey *et al.* (2003)
*Pristionchus pacificus*	V	L0CML2	AGWK**NS**I**RH**NL**SL**HSRF	Ogawa *et al.* (2011)
*Strongyloides stercoralis*	IVa	Q6WKW2	AGWK**NS**I**RH**NL**SL**HNRF	Massey *et al.* (2003)
*Meloidogyne hapla*	IVb	Contig353.frz3.gene4	QGWK**NS**I**RH**NL**SL**HSRF	Nematode.net; Opperman *et al.* (2008)
*Meloidogyne incognita*	IVb	Minc17526 (UPI00060F5D60)	WGWQ**NS**I**RH**NL**SL**HDCF	Meloidogyne genomic resources (INRA); Abad *et al.* (2008)
*Globodera pallida*	IVb	GPLIN_001276900	SGWK**NS**V**RH**NL**SL**NKCF	Gene DB
*Heterodera schachtii*	IVb	HS00253	QGWK**NS**I**RH**NL**SL**HSRF	Nematode.net
*Bursaphelenchus xylophilus*	IVb	H2DMI5	AGWK**NS**I**RH**NL**SL**HSRF	Kikuchi *et al.* (2011)
			**:**:******.. *	

The predicted residues involved in direct contact with DNA are highlighted in bold according to a previous analysis of the forkhead DNA-binding domain in DAF-16 (Obsil and Obsilova, 2011; Nakagawa *et al.*, 2013). Protein BLAST searches were performed using *C. elegans* DAF-16 proteins as query sequences. Multiple amino acid alignments were performed with Clustal Omega (Sievers *et al.*, 2011). The consensus symbols refer to fully conserved (*), strongly similar (:) and weakly similar (.) sequences. ‘Nematode clade’ refers to the five major phylogenetic groups within nematodes according to Blaxter (1998).

**Table 2:** Homology searches of SKN-1 in the phylum Nematoda for its core DNA-binding regions

Species	Nematode clade	Current annotation	Adjacent basic region	Core DNA binding region	Website/reference(s)
*Caenorhabditis elegans*	V	P34707	_452_QRKRG**R**QSKDEQL_464_	_506_**R**KI**RRR**G**KNK**V**AARTCR**Q**RR**_525_	Rupert *et al.* (1998), Choe *et al.* (2012*b*)
*Pristionchus pacificus*	V	H3EVC1	PRRRG**R**QSKDEQL	**R**KI**RRR**G**KNK**V**AARTCR**Q**RR**	Choe *et al.* (2012*b*)
*Strongyloides stercoralis*	IVa	SS01750	KKKAG**R**VSKDNEL	**R**NI**RRR**G**RNK**I**AAKKVR**I**NR**	Nematode.net; Choe *et al.* (2012*b*)
*Meloidogyne hapla*	IVb	Contig1686.frz3.gene3	KGKRG**R**RSKDDSL	**K**KI**RRR**G**RNK**L**AARKCR**D**RR**	Nematode.net
*Meloidogyne incognita*	IVb	Minc09034 (MI04199)	KGKRG**R**RSKDDSL	**K**KI**RRR**G**RNK**L**AARKCR**D**RR**	Nematode.net; Choe *et al.* (2012*b*)
*Globodera pallida*	IVb	GPLIN_000599400	KCKRG**R**KSKDNSL	**K**KI**RRR**G**RNK**F**AAQKCR**E**RR**	Gene DB
*Heterodera schachtii*	IVb	HS01483	KSKRG**R**KSKDNSL	**K**KI**RRR**G**RNK**L**AAQKCR**E**RR**	Nematode.net; Choe *et al.*, 2012b
*Bursaphelenchus xylophilus*	IVb	BUX.s01653·203	QRKRG**R**QSKDEQL	**K**KI**RRR**G**RNK**V**AARKCR**E**RR**	Nematode net; Kikuchi *et al.* (2011)
			: ** ***:.*	::*****:**.**:. * .*	

The predicted residues involved in direct contact with DNA are highlighted in bold according to a previous analysis of the Cap’n’collar (CNC) DNA-binding domain and adjacent basic region (BR) in SKN-1 (Rupert *et al.*, 1998, Choe *et al.*, 2012*b*; Blackwell *et al.*, 2015). Protein BLAST searches were performed using *C. elegans* SKN-1 proteins as query sequences. Multiple amino acid alignments were performed with Clustal Omega (Sievers *et al.*, 2011). The consensus symbols refer to fully conserved (*), strongly similar (:) and weakly similar (.) sequences. ‘Nematode clade’ refers to the five major phylogenetic groups within nematodes according to Blaxter (1998).

### Targets of SKN-1 and DAF-16 during stress response in nematodes

As mentioned previously, SKN-1 and DAF-16, together with their respective orthologous genes in other animals, are the subject of numerous investigations focusing on development, ageing, lifespan extension and immunity (Shivers *et al.*, 2008; Kenyon, 2010; Martins *et al.*, 2016). As the gene function of DAF-16 and SKN-1 has been extensively examined, we have paid greater attention to their central role during the management of cellular stress responses and cytoprotection (Shore and Ruvkun, 2013) and their potential link to PPNs. Several studies have generated data on global gene expression of *daf-16* and *skn-1*. For instance, Murphy *et al.* (2003) published transcriptome profiles of *daf-2*, *age-1* and *daf-16*/*daf-2* mutants in comparison with those of wild-type animals and by combining the mutants with RNAi screens targeting the DAF-2/IGF-1 and DAF-16/IGF-1 pathways. In addition, transcriptome analyses have been performed in wild-type animals that were treated with *skn-1* dsRNA under normal or oxidative conditions (Park *et al.*, 2009). Other studies based on promoter analysis, epistasis, chromatin immuno-precipitation (ChIP) assays and/or proteomic analysis have supported these findings and enriched the targets of these genes (Oliveira *et al.*, 2009; Niu *et al.*, 2011; Glover-Cutter *et al.*, 2013; Tepper *et al.*, 2013; Tullet, 2015). Moreover, their orthologous genes in mammals and invertebrates have been fully elucidated (Hayes and Dinkova-Kostova, 2014; Webb and Brunet, 2014; Martins *et al.*, 2016; Webb *et al.*, 2016). DAF-16 and SKN-1 are responsible for gene activation of up to 500 and 846 genes, respectively, with some overlapping targets. Several functional gene groups from these interactions are implicated in the antioxidant pathway, the detoxification pathway and the unfolding response pathway (Espinosa-Diez *et al.*, 2015; Klotz *et al.*, 2015; Pajares *et al.*, 2015).

#### The nematode antioxidant pathway.

In *C. elegans*, DAF-16 and SKN-1 act in concert to up-regulate the expression of two catalases (*CTL-1* and *CTL*-*2*) and two superoxide dismutases (*SOD-1* and *SOD-3*). Consistently, *SOD-3* and *CTL* genes are highly expressed in dauer larvae and *daf-2* mutants (Vanfleteren and de Vreese, 1995; Honda and Honda, 1999). SKN-1 also up-regulates expression of two genes that encode glutathione peroxidases. DAF-16 and SKN-1 are also associated with the up-regulation of the *PRDX-2* gene that encodes peroxiredoxin in *C. elegans* (Oláhová and Veal, 2015). Comparative genomics studies have enabled ROS-scavenging enzymes to be identified in PPNs (Abad *et al.*, 2008; Opperman *et al.*, 2008; Bird *et al.*, 2009). Their role in parasitism has been experimentally validated using proteomic and molecular approaches (Shinya *et al.*, 2013). Additionally, secretomes from *M. incognita* and *B. xylophilus* have revealed several proteins linked to the antioxidant pathway (Bellafiore *et al.*, 2008; Rosso *et al.*, 2009; Shinya *et al.*, 2013; Mitchum *et al.*, 2013). These studies have found mitochondrial and cytoplasmic SODs and CTLs in *M. incognita* and *B. xylophilus* (Bellafiore *et al.*, 2008; Shinya *et al.*, 2013). Moreover, the virulence of *B. xylophilus* is correlated with the degree of catalase expression and, by extension, its resistance to oxidative stress (Vicente *et al.*, 2015). The PPN secretome also comprises two other relevant ROS-scavengers, GPX and PRX enzymes that are implicated in the regulation of a defensive oxidative burst from the plant. Seven *PRX* genes have been found in the genome of *M. incognita* (Abad *et al.*, 2008). *PRX* expression occurs in the exophytic phase and reaches a higher expression level during RKN parasitism (Dubreuil *et al.*, 2011). Here, *PRX* gene silencing affects nematode parasitism, suggesting that PRX promotes oxidative stress resistance in PPNs. Li *et al.* (2016) have also recently reported a cytoprotective role of PRX in *B. xylophilus* during the infestation of whitebark pine (*Pinus bungean*). These results showed that PRXs from *M. incognita* and *B. xylophilus* exhibit strong antioxidant activities in parasitic stages. The role of these antioxidant enzymes is similar to that observed in *C. elegans* during biotic and abiotic stress and ageing (Ewbank, 2006; Golden and Melov, 2007).

#### The nematode detoxification pathway.

The xenobiotic/endobiotic detoxification pathway is one line of defence mediated by SKN-1 and/or DAF-16 in nematodes (Depuydt *et al.*, 2013; Blackwell *et al.*, 2015). Xeno- and endobiotics refer to toxic molecules produced exogenously or endogenously. The detoxification pathway is conserved across the animal, plant and fungal kingdoms (Blokhina *et al.*, 2003; Xu *et al.*, 2005; Lindholm *et al.*, 2006). In a general manner, the host plant can produce a wide diversity of secondary metabolites with potential effects on PPNs (Ohri and Pannu, 2010; Caretto *et al.*, 2015). The toxic properties of phytoalexins that are secreted in response to nematode infections are specifically seen as xenobiotics (Chitwood, 2002). A large group of these metabolites are derived from phenolic compounds. For example, we previously mentioned that in the *R. similis*–*Musa* spp. interaction, the phytoalexin nematicide (phenalenone) is detected at high concentrations in the lesions induced by the parasite (Wuyts *et al.*, 2007; Hölscher *et al.*, 2014). The best detoxification mechanism characterized in nematodes is in *C. elegans*, with studies regarding ageing and immune responses (Gems and Doonan, 2008; Shivers *et al.*, 2008; Engelmann and Pujol, 2010). The SKN-1-dependent detoxification pathway has previously been proposed to play an important role in PPNs to disarm the plant’s xenobiotic metabolism that belongs to its defensive arsenal (Kikuchi *et al.*, 2011; Choe *et al.*, 2012*b*). This pathway involves three detoxification enzyme systems that are essentially hosted in the endoplasmic reticulum (ER) (Xu *et al.*, 2005; Lindholm *et al.*, 2006; Blackwell *et al.*, 2015). The phase 1 detoxification system involves enzymes from the cytochrome P450 (CYP) and short chain dehydrogenase (SDR) families, whereas phase 2 concerns enzymes from two other families, i.e. glutathione *S*-transferases (GSTs) and UDP-glucuronosyl transferases (UGTs). These enzymes orchestrate the inactivation of xeno- and endobiotics by the suppression or addition of functional groups. During phase 3, conjugated toxins are exported from cells by dedicated transporters, such as the ATP-binding cassette (ABC) transporter family. A transcriptome analysis revealed a high gene expression level of *GST-1* during plant parasitism by *M. incognita* (Dubreuil *et al.*, 2007; Bellafiore *et al.*, 2008), with GST-1 being secreted from oesophageal secretory glands, suggesting a functional role in nematode feeding sites. Additionally, Shinya *et al.* (2013) also detected a total of 12 antioxidant proteins, including two GSTs (GST-1 and GST-3), based on a secretome analysis of *B. xylophilus*. Interestingly, aurovertin D, a secreted metabolite isolated from the nematophagous fungus *Pochonia clamydosporia*, exhibits strong nematicidal properties against *C. elegans* and *M. incognita* (Wang *et al.*, 2015*a*). Epistasis analyses of *daf-2* and *daf-16* mutants in *C. elegans* have shown that these mutants are resistant and hypersensitive, respectively, to aurovertin D treatment. Probably, the *daf-16* mutant is unable to detoxify this molecule. As well as exobiotics, the formation of endobiotics should also be taken into account. During host infection, cellular components of the nematode are exposed to reactive species, generating protein carbonylation, lipid peroxidation or oxidative by-products of nucleic acids. For example, reactive species produced in the plant cell wall can cause lipid peroxidation of membranes in PPNs. Peroxidized lipids are also targets for glutathione lipid hydroperoxidases and GSTs from the detoxification pathway (Gems and Doonan, 2008). Finally, one role of the detoxification pathway in nematode parasitism is to protect the animal from nematicidal compounds produced by the host immunity. For this reason, the generation of xenobiotic inhibitors has been proposed as a strategy for suppressing parasitic nematodes (Choe *et al.*, 2012*b*).

#### The nematode unfolding protein pathway.

Other genes are up-regulated by SKN-1 and DAF-16 to support the maintenance of proteostasis, and these include heat shock proteins (HSPs) that stabilize protein folding by direct interactions (Murphy *et al.*, 2003). For example, DAF-16 enhances *HSP* gene expression (*HSP-16*, *HSP-12·6* and *SIP-1*) (Hsu *et al.*, 2003). Besides the role in the antioxidant pathway, GST enzymes are capable of preventing the cysteine oxidation in proteins. In addition, thioredoxins (THXs) and protein disulfide isomerases (PDIs) also participate in protein maintenance by reducing or rearranging disulfide bond formation. SKN-1 up-regulates the expression of one gene that encodes THX and two that encode PDI (Glover-Cutter *et al.*, 2013). Proteomic analysis also found these protein families in the secretome of PPNs (Bellafiore *et al.*, 2008; Rosso *et al.*, 2009; Shinya *et al.*, 2013; Mitchum *et al.*, 2013). Another aspect of protein maintenance concerns a link between the detoxification pathway and the ER, which is associated with the oxidative stress response. Inhibition of the detoxification pathway increases oxidative damage and thus amplifies oxidative stress. DAF-16 and SKN-1 orchestrate the redox balance between the ER and cytoplasmic redox status, allowing the adaptation of cellular homeostasis during stress (Ron and Walter, 2007; Safra *et al.*, 2014; Cominacini *et al.*, 2015). Moreover, the implication of SKN-1 in modulation of the unfolding protein response (UPR) has been reported. A ChIP assay on SKN-1 revealed several important genes related to the UPR signalling pathway that are positively regulated by SKN-1 under oxidative stress (Niu *et al.*, 2011). The UPR signalling pathway prevents misfolded proteins by triggering protein maintenance and slowing global transcriptional activity. This molecular mechanism affects the traffic jam of proteins in the ER (Glover-Cutter *et al.*, 2013). Moreover, UPR maintenance homoeostasis and DAF-16-regulated genes have been associated with the secretion of proteins that were implicated in detoxification and cytoprotection (Shore and Ruvkun, 2013; Safra *et al.*, 2014).

**Fig. 2.**
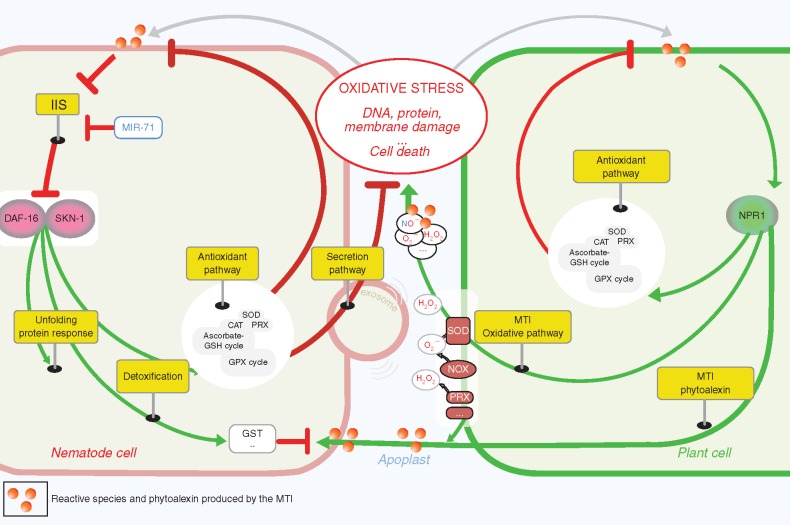
How do plant-parasitic nematodes alleviate the stress of an apoplast ‘on fire’? A model is proposed to explain the DAF-16 and SKN-1 functions that were orchestrated within different cellular pathways to resist the release of toxic compounds (reactive species and phytoalexins) by the plant cell early in infection. In this model, the nematodes sense the physiological state of the plant cell by detecting reactive species, and they generate an adapted response. The findings illustrated in this figure are based primarily on multi-omic resources from plant-parasitic nematodes and functional genomics data from the nematode model system *C. elegans*. The plant defence response activates the oxidative pathway, leading to the release of reactive species in the apoplast. At the same time, the plant activates its antioxidant pathway to protect the plant cell from oxidative damage. Secondary metabolism is modulated to produce phytoalexins, which represent xenobiotics to the nematode cell. Additionally, the perception of reactive species leads the nematode cell to activate its oxidative stress response. In *C. elegans*, this pathway is orchestrated by DAF-16 and SKN-1, two transcription factors that are conserved in the animal kingdom. DAF-16 and SKN-1 are negatively regulated by the insulin/IGF-1 signalling (IIS) pathway and positively regulated by miR-71. As in the plant cell, the activation of the antioxidant pathways has a cytoprotective function. In parallel, ROS-scavenging enzymes can be secreted in the apoplast to mitigate the plant’s oxidative burst. The unfolding protein response (UPR) adapts cellular homeostasis and protects proteins directly from oxidative damage induced by oxidative stress. The detoxification pathway covers the phytoalexins produced by the plant cell to suppress their toxicity. GSH, glutathione; CAT, catalase; PER, peroxidase; PRX, peroxiredoxin; SOD, superoxide dismutase; GPX, glutathione peroxidase; SKN-1, skinhead transcription factor-1; DAF-16, dauer formation 16; DAF-12, dauer formation-12; NPR1, non-pathogenic related protein-1; NOX, NADPH oxidase; MTI, MAMP-triggered immunity; MIR-71, micro RNA-71; IIS, insulin IGF1 signalling.

SKN-1 and DAF-16 regulate the antioxidant, the UPR and the detoxification pathways during cellular events. They are important components at the crossroads of several cellular pathways, more specifically involved in oxidative stress responses. Numerous enzymes under the control of DAF-16 and SKN-1 are often detected in the secretome of PPNs in the early stages of parasitism, as cited previously. Regardless of whether *C.elegans DAF-16* and *SKN-1* orthologues in PPNs may be crucial in the early stages, they represent a potential defensive strategy against host immunity (Fig. 2). However, activation of the antioxidant, detoxification and UPR pathways strongly consumes NADPH and GSH and represents a huge cost in terms of energy and reducing power. Over time, this cost is critical for the cellular redox balance towards oxidative stress and, consequently, generates a poisonous environment for the nematode leading to intoxication. To defend itself, the small animal may deploy an offensive strategy by curtailing locally the production of plant defensive compounds. This requires the secretion of effectors capable of controlling plant immune responses. We mentioned previously that genes encoding TTL proteins have been identified as effector-modulating host immunity factors in APNs and PPNs (Goverse and Smant, 2014; Mantelin *et al.*, 2015). Curiously, numerous transthyretin-like proteins in *C. elegans* are significantly up-regulated in DAF-16- and IIS-dependent manners (Depuydt *et al.*, 2013) and are thus associated with innate immunity against microbial nematicides (Treitz *et al.*, 2015). The TTL gene family may be an example of potential nematode effectors that are secreted in response to the oxidative stress response induced by its host plant.

### Regulatory elements of DAF-16 and SKN-1 in nematodes

DAF-16 and SKN-1 contribute to the orchestration of several adaptive cellular pathways in response to various environmental stimuli (Schindler and Sherwood, 2014; Blackwell *et al.*, 2015). In *C. elegans*, the effects of aspirin and SA on stress responses have been assessed (Ayyadevara *et al.*, 2013; Wan *et al.*, 2013). In a survival assay, aspirin (0·5–1 mm) and SA (1 mm) were administered through the nematode medium, leading to an increase of 1·5- to 3·5-fold in the expression of genes encoding SOD, CTL and GST proteins *(*Ayyadevara *et al.*, 2013). In contrast, *daf-16* mutant fails to up-regulate *SOD* and *CTL* genes under SA application, implicating DAF-16 in the SA-mediated activation of these genes. The nematode lifespan then increases by 12–30 % when small animals are treated with aspirin or SA upon exposure to 5 mm H_2_O_2_. This finding correlates activation of the antioxidant pathway with the nematode’s capacity to resist harsh oxidative stress in a DAF-16-dependent manner. Moreover, protein aggregation decreases under oxidative stress upon SA treatment underlying the role of this signalling molecule within the UPR pathway in *C. elegans.* In fact, SA, Me-SA and its derivatives (called salicylates) are ancient signalling molecules that are found across taxa to have physiological impacts in the animal kingdom (Klessig *et al.*, 2016). In the pine–*B. xylophilus* interaction, SA elevation is significantly detected at early stages of infection (Li *et al.*, 2016). Moreover, ascaroside pheromones from diverse PPNs were found to activate MTI and especially genes from the SA pathway in host plants (Manosalva *et al.*, 2015). Interestingly, a screen of plant exudates revealed that SA is an attractant for *M. incognita*, suggesting an impact of the signalling molecule on nematode physiology and behaviour (Wuyts *et al.*, 2006). It is tempting to speculate that DAF-16 or SKN-1 pathways in PPNs could be triggered by SA perception. In such cases, sensing SA to activate an oxidative stress response might represent an advantage for PPNs to resist the plant immune system in the early stages of infection.

Downstream of the stimulus perception, oxidative stress response is modulated by evolutionary conserved signalling pathways in the animal kingdom (Shivers *et al.*, 2008; van der Hoeven *et al.*, 2011; Shore and Ruvkun, 2013; Blackwell *et al.*, 2015; Klotz *et al.*, 2015; Penkov *et al.*, 2015). In a general manner, the regulation of DAF-16 and SKN-1 involves an intricate network of post-translational modifications and protein–protein interactions. Interestingly, some important regulatory elements cited below have been identified in PPNs and a few have been validated for their implication in plant parasitism by an RNAi approach (Fig. 1). Moreover, *C. elegans* mutants affected in these genes have shown a contrasting response to oxidative stress. As discussed previously, the IIS pathway plays a pivotal role in stress responses. Insulin perception by the DAF-2 receptor activates the phosphoinositide-dependent kinase-1/serine-threonine kinase (PDK-1/AKT) phosphorylation cascade that prevents nuclear *trans*-localization of DAF-16 and SKN-1 (Lin *et al.*, 2001; Henderson and Johnson, 2001). Reciprocally, SKN-1 negatively regulates IIS through a positive feedback loop (Oliveira *et al.*, 2009). Futhermore, the Akt phosphorylation sites in DAF-16 mediate binding of the 14-3-3 scaffolding proteins and contribute to its sequestration within the cytoplasm (Li *et al.*, 2007). At a post-transcriptional level, miR-71 silences the gene expression of *AGE-1* and *AKT*, positioning this miRNA as an inhibitor of the IIS pathway (de Lencastre *et al.*, 2010; Boulias and Horvitz, 2012). In addition, the p38 mitogen-activated protein kinase (MAPK) pathway also plays an important function in orchestration of the oxidative stress response. Exogenous ROS exposure or an endogenous production by dual oxidase 1 (DUOX1) is associated with SKN-1 translocation in the nucleus via the p38 MAPK pathway (Inoue *et al.*, 2005; van der Hoeven *et al.*, 2011). To our knowledge, some of these genes from the IIS and p38 MAPK pathways have been explored for their physiological and virulence roles in PPNs. Genome sequencing of *M. incognita* and *B. xylophilus* revealed *C. elegans* genes orthologous to 14-3-3, *PDK-1*, *AGE-1* and *AKT-1*&*2* (Abad *et al.*, 2008; Shinya *et al.*, 2010; Kikuchi *et al.*, 2011; Liu *et al.*, 2011). Silencing of *DUOX1* and *MPK-1* genes in *M. incognita* compromised nematode parasitism in tomato plants (Charlton *et al.*, 2010; Dong *et al.*, 2016). Interestingly, deep sequencing analysis from *M. incognita* has identified miR-71 as one of the most frequently produced miRNAs at the pre-infective J2 stage and in eggs (Wang *et al.*, 2015*b*; Zhang *et al.*, 2016). Beyond the central role of the IIS and p38 MAPK pathways for the oxidative stress response orchestration, other post-translational modifications and protein interactions of DAF-16 and SKN-1 might potentially be of interest in the understanding of plant–nematode parasitism (Yen *et al.*, 2011; Riedel *et al.*, 2013; Benayoun *et al.*, 2015; Blackwell *et al.*, 2015).

Furthermore, the gene expression profile of DAF-16 and SKN-1 in *C. elegans* has been essentially reported in the intestinal cells but also in hypodermis and neural cells (Libina *et al.*, 2003; Chávez *et al.*, 2007; Murphy *et al.*, 2007; Paek *et al.*, 2012). Nevertheless, their expression can vary according to the physiological context. For example, nematode infection by a fungal pathogen is associated with the expression and activation of DAF-16 in the epidermal tissues in contact with the pathogen (Zou *et al.*, 2013). Similarly, DAF-16 and SKN-1 activation can drastically increase in intestinal cells during exposure to xenobiotics or pathogens (Chávez *et al.*, 2007; van der Hoeven *et al.*, 2011; Papp *et al.*, 2012). More in depth, *DAF-16* is subjected to a positive feedback regulation in intestinal cells following the model known as ‘FOXO-to-FOXO’ (Murphy *et al.*, 2007). In other words, a systemic signal amplifying *DAF-16* gene expression and its activation is propagated within intestinal cells and throughout other tissues in the animal. A similar mechanism has also been mentioned for *SKN-1* (Staab *et al.*, 2013). The cell-to-cell communication comprises miR-71 expression in the neuronal system and is linked to DAF-16 activation in the intestinal cells (Boulias and Horvitz, 2012). The DAF-16 activity in intestinal cells influences the systemic signalling by coordinating downstream gene expression in a tissue-specific way, such as *SOD-3* expression in the epidermis and muscles. (Libina *et al.*, 2003). In PPNs, epidermis and intestine are two organs exposed to plant xenobiotics and ROS released in the extracellular matrix. PRX immunolocalization in *M. incognita* has been observed in the hypodermis and the pseudocoelom surrounding the stylet and amphidial pouches, which suggests a protective role when locally in contact with the feeding cells (Dubreuil *et al.*, 2011). By *in situ* hybridization, Vicente *et al.* (2015) have shown that *CTL-1* and *CTL-2* genes are expressed in the intestinal cells of *B. xylophilus*. The orchestration of antioxidant responses probably involves a complex signalling network between various organs within the nematode. Following Murphy’s model (Murphy *et al.*, 2007) in *C. elegans*, the nematode intestine is an organ with a fundamental role in the DAF-16 and SKN-1 pathways. The systemic signalling that is propagated from the intestinal cells seems to resonate with other organs, orchestrating a coherent oxidative stress response.

## CONCLUDING REMARKS

Given the considerable economic impact of PPN pathogens on global crop yield (Chitwood, 2003), the development of management strategies for disease control requires particular attention. Recent research has focused on either plant breeding for engineering durable disease resistance to PPNs or on identification of effectors and their role in successful parasitism. With advances in whole genome sequencing in both the model species *C. elegans* and parasitic nematode species, significant progress has been made in identifying molecular players that modulate important biological processes. The present review investigates events prior to PPN establishment of successful infection and how these nematodes are able to manage the stressful environment imposed by the host plant throughout the parasitic life cycle. We portray the putative role of PPN orthologues of *C.elegans* DAF-16 and SKN-1 in sensing and tackling the first line of host defence. DAF-16 and SKN-1 are ancient transcription factors conserved across the animal kingdom, which play essential roles in nematode development and environmental adaptation. We propose that they might also play an important role in PPNs in counteracting oxidative stress conditions produced by the host plant throughout invasion and migration.

Hub proteins have been defined as conserved proteins that are highly connected within cellular pathways and act as strong regulators of organism development and environmental adaptation (Dietz *et al.*, 2010; Grants *et al.*, 2015). *DAF-16* and *SKN-1* share these features and can be considered as hub proteins (Schindler and Sherwood, 2014; Webb and Brunet, 2014). Such proteins are often identified as cellular targets by pathogens (Mukhtar *et al.*, 2011; Schleker and Trilling, 2013). In *C. elegans*, as in plants, the immune system also involves a first line of protection against pathogens, resulting from the activation of an oxidative burst (Engelmann and Pujol, 2010; Papp *et al.*, 2012) and simultaneous activation of cellular preservation mechanisms regulated by DAF-16 and SKN-1 (Shore and Ruvkun, 2013; Espinosa-Diez *et al.*, 2015; Klotz *et al.*, 2015). Finally, in plant–nematode interactions, the host triggers an oxidative burst and the PPN has to fight against it. If SKN-1 and DAF-16 orchestrate oxidative stress responses in *C. elegans*, probably PPN orthologues could do the same following a partially similar mechanism.

More generally, it was shown that DAF-16 and the IIS pathway are at the crossroads of redox and metabolism in *C. elegans* (Penkov *et al.*, 2015). This finding supports the hypothesis that there are developmental checkpoints in *C. elegans* in accordance with the model ‘*should I stay/should I go*’ (Murphy and Hu, 2013; Schindler and Sherwood, 2014). A hypothesis would be that the IIS pathway comprising *C. elegans DAF-16* orthologues in PPNs takes part in the ‘*go/no-go checkpoints*’ model during nematode parasitism and its exophytic stage (Goverse and Smant, 2014; Schindler and Sherwood, 2014). Therefore, it would be interesting to establish comparisons between the ‘*go/no-go checkpoint*’ model from plant–nematode interactions and the ‘*should I stay or should I go*’ model derived from the IIS established in *C. elegans*.

Many current nematode control measures are based on nematicides. These agrochemicals can have toxic effects on human health and also be limited to only reduced nematicidal activity at increased soil depths, where nematode populations can occur. A common alternative biotechnological approach for nematode control, which is based on engineering plant resistance, relies on the induction of RNAi silencing to target essential nematode genes for nematode development, reproduction, metabolism or stress response (Rosso *et al.*, 2009; Li *et al.*, 2011; Dutta *et al.*, 2015; Rehman *et al.*, 2016). The high connectivity and important functions of DAF-16 and SKN-1 in the nematode response against oxidative stress and other cellular pathways make them attractive upstream transcription factor targets for RNAi, and represent an alternative to RNAi pyramiding strategies, as several essential genes may be silenced simultaneously. Down-regulation of DAF-16 and SKN-1 probably affect not only the expression of genes encoding ROS scavengers and detoxification enzymes, but also PPN cell functions during their exophytic stage, particularly during abiotic and biotic stress.

In conclusion, genomics data for the *C. elegans* model system, together with those for PPNs, are enabling advances in understanding adaptation mechanisms involved in nematode parasitism, in addition to how PPN effectors modulate plant cellular pathways. Unravelling of the molecular mechanisms involved in PPNs for managing plant defence responses is furthering our general understanding of the pathosystem. From a biotechnological point of view, this may offer the possibility for identification of new targets in the development of durable resistance to PPNs in plants.
